# Genotype-Specific Clinical Response to a Lactose-Free Diet in Adult Patients Evaluated for Lactose Intolerance: Implications for Clinical Assessment

**DOI:** 10.3390/nu18172764

**Published:** 2026-08-24

**Authors:** Roxana Elena Mirică, Claudia Simona Stefan, Alexandru Nechifor, Alina Breazu, Laura Maria Batis, Ioana Soare

**Affiliations:** 1 Faculty of Medicine, “Carol Davila” University of Medicine and Pharmacy, 020021 Bucharest, Romania; 2 Regina Maria, Private Healthcare Network, 077190 Bucharest, Romania; 3 Department of Pharmaceutical Sciences, Faculty of Medicine and Pharmacy, “Dunarea de Jos” University of Galati, 800008 Galati, Romania; claudia.stefan2009@ugal.ro; 4 Department of Individual Sports and Physiotherapy, Faculty of Physical Education and Sport, “Dunarea de Jos” University of Galati, 800008 Galati, Romania; alexandrunechiformed@yahoo.com; 5 Public Health and Health Services Management, 021253 Bucharest, Romania; dr_alinabreazu@yahoo.com; 6 Department of Geriatrics, Faculty of Medicine, “Titu Maiorescu” University, 040051 Bucharest, Romania; laurabatis@yahoo.com; 7 Department of Social Insurance Medicine, “Titu Maiorescu” University, 040051 Bucharest, Romania; ioana_soare@yahoo.com

**Keywords:** lactose intolerance, LCT genotypes, lactase non-persistence, lactose-free diet, symptom improvement, adult population, genetic testing, medical assessment

## Abstract

**Background**: Lactose intolerance is a common gastrointestinal condition with variable clinical expression, often requiring both clinical and genetic evaluation. While genetic testing identifies genetic predisposition to lactase persistence or non-persistence, the relationship between specific LCT genotypes and clinical response to dietary intervention remains incompletely understood. **Aim**: To assess the association between LCT genotypes (CC, CT, TT) and symptom improvement following a lactose-free diet in an adult population and to explore the implications for clinical assessment. **Methods**: A retrospective observational study was conducted including 538 adult patients evaluated for suspected lactose intolerance. Genotype distribution and its association with clinical response to a lactose-free diet were analyzed. Group comparisons were performed using chi-square and non-parametric tests, and logistic regression analysis was used to identify genotype-specific predictors of symptom improvement. **Results**: The CC genotype was the most prevalent (70.8%), followed by CT (22.9%) and TT (6.3%). No significant differences were observed between genotype groups regarding age, sex, or area of residence. A significant association was identified between LCT genotype and clinical response to lactose restriction (*p* < 0.001). CT carriers demonstrated the highest rate of symptom improvement (70.7%), followed by CC (43.8%) and TT (17.6%). Logistic regression analysis confirmed higher odds of improvement among CT carriers (OR = 11.28, 95% CI: 4.30–29.56, *p* < 0.001) and CC carriers (OR = 3.64, 95% CI: 1.47–9.00, *p* = 0.005) compared with TT individuals. **Conclusions**: LCT genotype was significantly associated with clinical response to a lactose-free diet. These findings suggest that genetic variation may contribute to differences in symptom response to dietary lactose restriction and support the use of genotypic information as a complementary component of clinical assessment. However, the observed variability in clinical outcomes across genotype groups highlights the multifactorial nature of lactose intolerance and indicates that genetic predisposition alone does not fully explain symptom expression or dietary response.

## 1. Introduction

Lactose intolerance is a frequent gastrointestinal condition resulting from reduced lactase activity, leading to impaired digestion of lactose and subsequent gastrointestinal symptoms. Its clinical presentation is often variable and nonspecific, including bloating, abdominal pain, and diarrhea, which may overlap with other functional gastrointestinal disorders [1,2]. Importantly, lactose malabsorption does not invariably result in clinically significant lactose intolerance, highlighting the multifactorial nature of symptom generation.

Lactose intolerance affects a substantial proportion of the global population, although its prevalence varies considerably according to ethnic and geographic background. Lactase non-persistence represents the physiological pattern in most adult populations worldwide, while lactase persistence is more frequently observed in Northern European populations due to genetic adaptation associated with dairy consumption. Despite its high prevalence, lactose intolerance remains underdiagnosed or inconsistently evaluated in clinical practice, partly because gastrointestinal symptoms are nonspecific and frequently overlap with other digestive disorders. In addition, self-diagnosis and empiric dietary restriction are increasingly common, potentially leading to unnecessary long-term avoidance of dairy products and adverse nutritional consequences [1,3].

Genetic lactase non-persistence, primarily associated with polymorphisms regulating LCT gene expression [4,5], represents a key mechanism underlying lactose malabsorption and may contribute to the development of lactose intolerance in susceptible individuals. The C/T-13910 polymorphism located upstream of the LCT gene is the most extensively studied genetic variant associated with lactase persistence in European populations. Several recent studies have investigated the relationship between LCT polymorphisms, lactose malabsorption, and gastrointestinal symptom severity, with heterogeneous findings across different populations. While some reports demonstrated a relatively strong correlation between lactase non-persistence genotypes and symptom development, others highlighted substantial variability in clinical expression, suggesting that additional environmental and host-related factors may contribute to symptom generation [6]. The distribution of LCT genotypes—commonly categorized as CC, CT, and TT—reflects varying levels of lactase activity, ranging from lactase non-persistence to persistence. Individuals with the CC genotype are typically associated with lactase non-persistence, whereas CT and TT genotypes are generally linked to lactase persistence [7]. However, the relationship between genotype and clinical symptom expression is not always straightforward, as not all individuals with lactase deficiency develop clinically significant symptoms [6].

In clinical practice, the diagnosis of lactose intolerance is frequently based on symptom assessment and response to dietary lactose restriction, whereas the presence of lactose malabsorption may be evaluated using genetic or functional testing [8]. However, the subjective nature of gastrointestinal symptoms and their variability may limit the accuracy of clinical evaluation. In this context, genetic testing has emerged as a complementary tool, providing objective information regarding lactase persistence status [9]. Furthermore, symptom severity may be influenced by additional factors, including visceral hypersensitivity, gut microbiota composition, and coexisting functional gastrointestinal disorders [10,11].

Recent advances in nutritional genomics and personalized nutrition have emphasized the importance of individual genetic variability in modulating dietary responses and gastrointestinal symptom expression. Increasing evidence suggests that genotype-guided nutritional approaches may improve diagnostic precision and contribute to more individualized dietary recommendations in patients with food-related gastrointestinal disorders. In this context, the integration of genetic information into routine clinical assessment has gained growing interest as part of precision medicine strategies [12,13,14].

In addition to genetic predisposition, emerging research suggests that gut microbiota composition, intestinal adaptation, and visceral sensitivity may further influence lactose tolerance and symptom severity. These complex interactions may partly explain the heterogeneous clinical manifestations observed among individuals sharing similar LCT genotypes, highlighting the multifactorial nature of lactose intolerance and the limitations of symptom-based diagnosis alone [1,3].

Despite its increasing use, the clinical utility of genetic testing in predicting symptom response to a lactose-free diet remains incompletely defined. In particular, differences in clinical outcomes among specific genotypes (CC, CT, and TT) are not fully elucidated [3,15]. A better understanding of genotype-specific clinical responses may contribute to improved diagnostic accuracy and more individualized dietary recommendations in patients evaluated for lactose intolerance.

The growing interest in personalized nutrition and precision medicine has further increased the clinical relevance of genotype-guided dietary interventions. In recent years, several studies have emphasized the importance of integrating genetic, clinical, and nutritional data in the evaluation of food-related gastrointestinal disorders. Beyond symptom control, individualized dietary management may also help reduce unnecessary dietary restrictions and improve long-term nutritional balance and quality of life. Consequently, understanding the relationship between LCT genotypes and clinical response to lactose restriction may have broader implications not only for diagnostic assessment, but also for personalized nutritional counseling strategies [12,14,16].

Therefore, the aim of this study was to evaluate the association between LCT genotypes and symptom improvement following a lactose-free diet in an adult population. Additionally, the study explores the potential role of genotype-based evaluation in supporting a more objective and individualized approach to medical assessment.

## 2. Methods

### 2.1. Study Design and Population

A retrospective observational analytical study was conducted including 538 adult patients evaluated for suspected lactose intolerance between 2021 and 2024. All participants underwent genetic testing for lactase persistence as part of routine clinical assessment.

Only patients with complete data regarding LCT-13910 C/T genotype and documented clinical follow-up after dietary intervention were included in the analysis. Patients with incomplete records were excluded.

### 2.2. Genetic Analysis

Genomic DNA was extracted from peripheral blood samples. Genotyping of the LCT-13910 C/T polymorphism was performed using polymerase chain reaction (PCR)-based methods, including real-time PCR and PCR-restriction fragment length polymorphism (PCR-RFLP), according to validated and routinely used laboratory protocols for clinical genetic testing [17,18,19].

Genotypes were categorized as CC, CT, and TT, corresponding to lactase non-persistence (CC) and lactase persistence-associated phenotypes (CT and TT), respectively.

### 2.3. Dietary Intervention

Patients were instructed to follow a strict lactose-free diet consisting of the complete avoidance of foods containing lactose, including conventional milk and dairy products. Lactose-free alternatives were recommended whenever available. After approximately 8 weeks of dietary restriction, patients were advised that limited consumption of certain naturally low-lactose or aged cheeses could be considered on an individual basis, depending on symptom tolerance.

Information regarding the use of commercially available enzymatically hydrolyzed dairy products was not systematically recorded because of the retrospective design of the study.

### 2.4. Data Collection

Demographic and clinical data were collected from medical records, including age, sex, and area of residence (urban or rural).

Clinical evaluation was based on routine follow-up consultations performed approximately 8 weeks after initiation of a lactose-free diet. Dietary adherence was recommended following diagnosis of suspected lactose intolerance.

Symptom assessment was based on patient self-reported changes in gastrointestinal symptoms, including bloating, abdominal pain, diarrhea, and constipation, compared with baseline status prior to dietary intervention. Symptoms were considered a clinical manifestation of lactose intolerance rather than a direct indicator of genetic lactase non-persistence.

Clinical outcomes were categorized as complete improvement (complete resolution of gastrointestinal symptoms), partial improvement (reduction in symptom intensity without complete symptom resolution), or no improvement (persistence of symptoms despite dietary intervention). Due to the retrospective design of the study, no validated symptom severity scale was systematically applied, and dietary adherence was evaluated during routine clinical follow-up consultations rather than through standardized dietary assessment questionnaires.

### 2.5. Outcome Definition

The primary outcome was clinical response to a lactose-free diet, defined as patient-reported improvement in gastrointestinal symptoms following dietary lactose restriction.

During routine follow-up assessment, clinical outcomes were initially classified as complete improvement (complete resolution of symptoms), partial improvement (reduction in symptom severity without complete symptom resolution), or no improvement (persistence of symptoms despite dietary intervention).

For statistical analysis, patients were subsequently categorized into two groups:

- Improvement: complete symptom resolution following dietary intervention.
- Partial or no improvement: persistent symptoms or incomplete symptom resolution despite dietary restriction.

Due to the retrospective design of the study, no validated symptom severity scale was systematically applied.

### 2.6. Statistical Analysis

Continuous variables were tested for normality and are presented as mean ± standard deviation or median (interquartile range), as appropriate. Categorical variables are expressed as frequencies and percentages.

Comparisons between genotype groups (CC, CT, TT) were performed using the Kruskal–Wallis test for continuous variables and the chi-square test for categorical variables.

The association between LCT genotype and clinical response to a lactose-free diet was assessed using logistic regression analysis. Odds ratios (ORs) and 95% confidence intervals (CIs) were calculated, using the TT genotype as the reference category.

A *p*-value < 0.05 was considered statistically significant.

All statistical analyses were performed using IBM SPSS Statistics, version 26.0.

## 3. Results

### 3.1. Study Population and Genotype Distribution

A total of 538 adult patients with complete clinical and genetic data were included in the analysis. The mean age of the study population was 39.8 ± 11.0 years, and 67.5% were female. Most participants resided in urban areas (84.8%).

**Figure 1:**
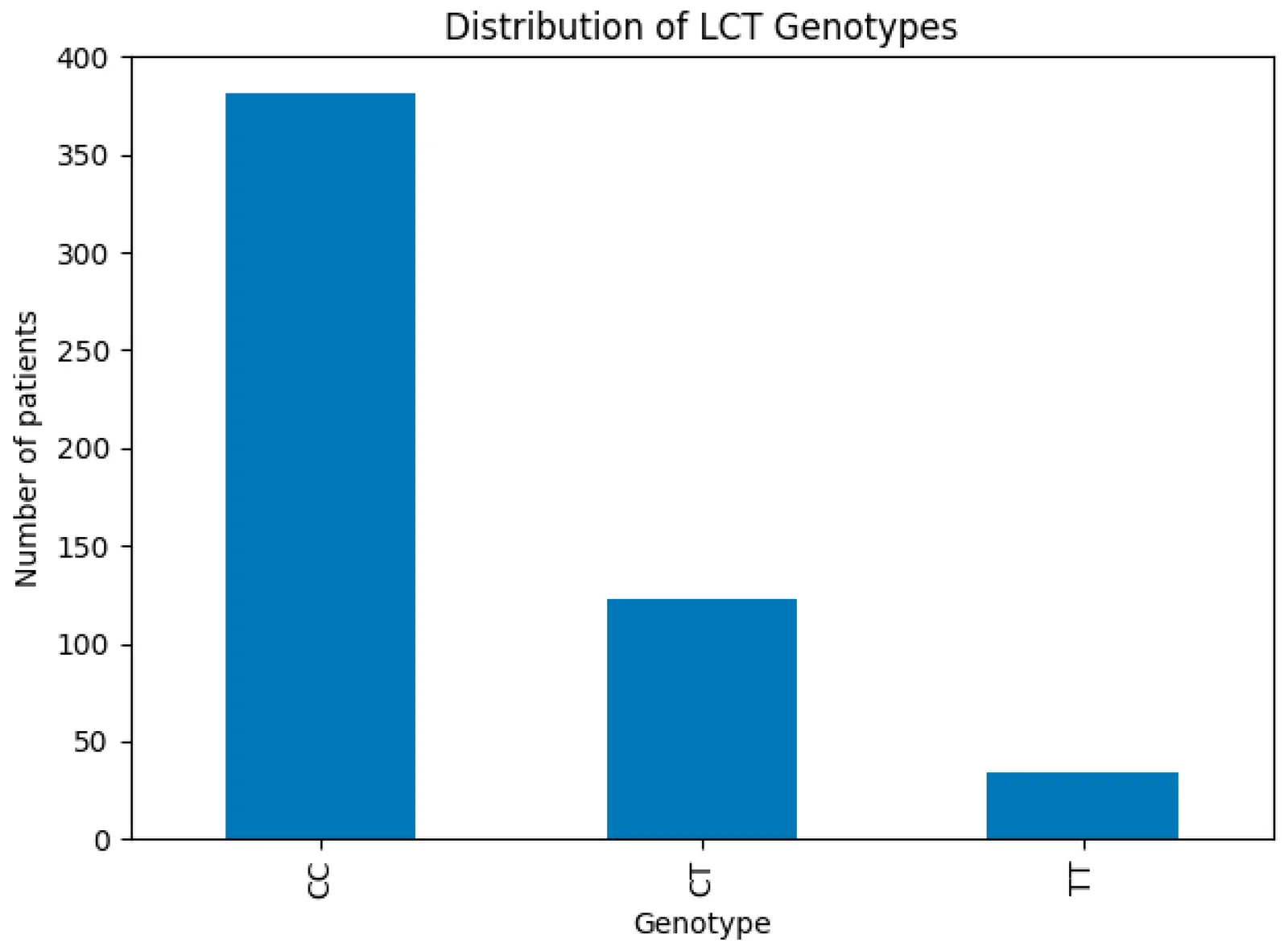
Distribution of LCT genotypes.

The CC genotype was the most frequently observed (381 patients, 70.8%), followed by CT (123 patients, 22.9%) and TT (34 patients, 6.3%). (Figure 1).

### 3.2. Baseline Characteristics Across Genotype Groups

No significant differences were observed between genotype groups regarding age (CC: 39.5 ± 10.9 years; CT: 40.7 ± 11.6 years; TT: 39.9 ± 10.6 years; Kruskal–Wallis test, *p* = 0.68).

Similarly, no statistically significant differences were identified in sex distribution (*p* = 0.73) or area of residence (urban vs. rural) (*p* = 0.29) across genotype groups (Table 1).

All patients presented with gastrointestinal symptoms at the baseline; therefore, no between-group comparison of baseline symptom prevalence was performed.

Baseline gastrointestinal symptoms included abdominal bloating, abdominal pain, and bowel habit disturbances (diarrhea and/or constipation). Abdominal bloating was reported by all participants, while abdominal pain was present in 252 patients (46.8%) and bowel habit disturbances in 124 patients (23.0%). The distribution of symptoms according to genotype is presented in Table 2.

### 3.3. Additional Gastrointestinal Investigations

Additional investigations were available for a subset of patients evaluated for alternative causes of gastrointestinal symptoms. Positive fecal Helicobacter pylori antigen tests were identified in 19 patients (3.5%), while positive Celikey screening tests suggestive of celiac disease were observed in 5 patients (0.9%) (Table 3). Among patients who underwent additional investigations based on clinical judgment, 19 tested positive for fecal *Helicobacter pylori* antigen, including 14 CC and 5 CT carriers, whereas no TT carrier tested positive. Celikey^®^ (Phadia AB, Uppsala, Sweden) screening was positive in five patients (four CC and one TT).

### 3.4. Clinical Response to Lactose-Free Diet

Clinical response to lactose restriction was evaluated after approximately 8 weeks of dietary intervention.

A significant association was observed between LCT genotype and clinical response to a lactose-free diet (χ^2^ test, *p* < 0.001) (Table 4).

**Figure 2:**
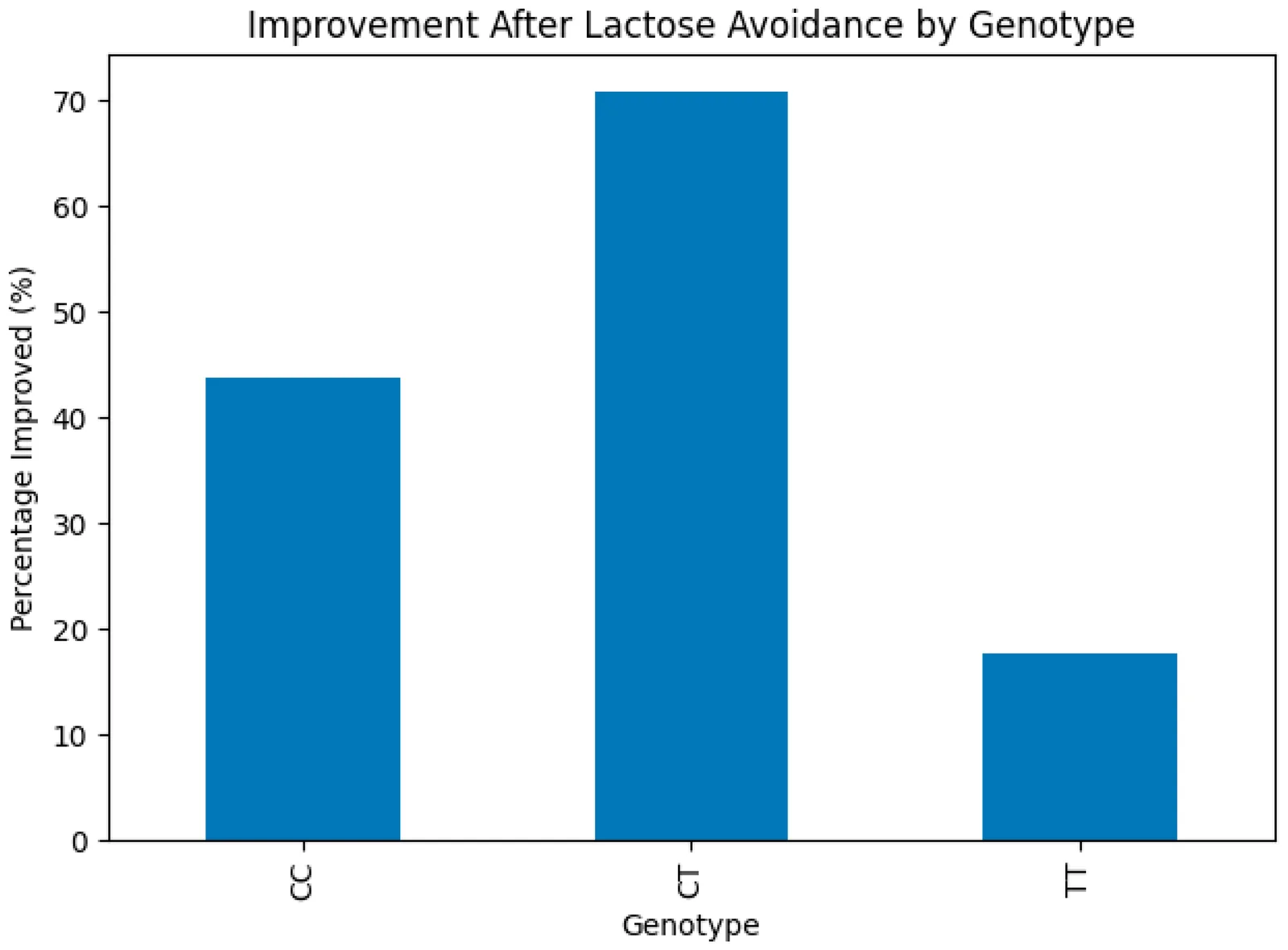
Improvement after lactose avoidance by genotype.

The highest rate of symptom improvement was observed in heterozygous CT carriers (70.7%), followed by CC carriers (43.8%), whereas TT individuals exhibited the lowest response rate (17.6%) (Figure 2).

### 3.5. Logistic Regression Analysis

Logistic regression analysis confirmed a significant association between LCT genotype and clinical response to lactose restriction.

Using the TT genotype as the reference category, CT carriers had significantly higher odds of symptom improvement (OR = 11.28, 95% CI: 4.30–29.56, *p* < 0.001). CC carriers also showed increased odds of clinical improvement compared with TT individuals (OR = 3.64, 95% CI: 1.47–9.00, *p* = 0.005).

### 3.6. Stratified Analysis (Mantel–Haenszel Method)

To further assess the robustness of the association between genotype and clinical response, a stratified analysis was performed using the Mantel–Haenszel method, adjusting for age—and sex—stratified analyses (Table 5).

The pooled Mantel–Haenszel odds ratio confirmed a significant association between LCT genotype and symptom improvement (OR_mh_ = 3.95; χ^2^MH = 6.70; *p* = 0.0097), consistent with the direction of effect observed in logistic regression analysis.

### 3.7. Summary of Findings

Overall, both unadjusted and adjusted analyses demonstrated a significant association between LCT genotype and clinical response to a lactose-free diet. The highest probability of symptom improvement was observed in CT carriers, followed by CC carriers, while TT individuals showed the lowest response rates.

## 4. Discussion

Within this study cohort, a significant association was observed between LCT genotype and clinical response to a lactose-free diet in adults evaluated for suspected lactose intolerance. Both unadjusted and adjusted analyses consistently showed that genetic variation at the LCT locus is associated with differences in symptom improvement following dietary lactose restriction.

A key finding of this study was that heterozygous CT individuals exhibited the highest rate of symptom improvement compared with both CC and TT genotypes. This association was confirmed by logistic regression analysis, which demonstrated significantly increased odds of clinical response in CT carriers relative to TT individuals, while CC carriers also showed a moderate but significant association with symptom improvement. One possible explanation for this finding is that heterozygous CT individuals retain partial lactase activity, allowing more efficient digestion of residual dietary lactose. Consequently, reducing lactose intake may be sufficient to substantially decrease the intestinal fermentation of undigested lactose and alleviate gastrointestinal symptoms. In contrast, individuals with the CC genotype generally experience long-standing lactase non-persistence, which may contribute to chronic intestinal adaptation, alterations in gut microbiota composition, and increased visceral hypersensitivity. These factors could partly explain why symptom improvement was less pronounced despite dietary lactose restriction. However, these mechanisms remain hypothetical, as gut microbiota composition, visceral sensitivity, and intestinal adaptation were not directly assessed in the present study [6,18,19]. The observation that a proportion of TT carriers also reported symptom improvement further illustrates the multifactorial nature of gastrointestinal symptoms. Because the TT genotype is generally associated with lactase persistence, symptom improvement in these individuals may reflect factors other than lactose malabsorption, including placebo effects, spontaneous symptom fluctuation, dietary modifications beyond lactose restriction, or coexisting functional gastrointestinal disorders. These findings reinforce that genetic testing should complement, rather than replace, comprehensive clinical evaluation when assessing patients with suspected lactose intolerance.

From a biological perspective, these findings may reflect inter-individual variability in lactase expression and intestinal adaptation to lactose intake. The C/T-13910 polymorphism located upstream of the LCT gene is strongly associated with lactase persistence in adulthood, although genotype–phenotype correlations are not absolute [20,21,22,23,24]. In European populations, the TT genotype is generally associated with lactase persistence, whereas the CC genotype is associated with lactase non-persistence [25,26,27]. However, intermediate phenotypes, variable lactase enzymatic activity, and differences in gastrointestinal sensitivity to dietary lactose have been described, particularly among heterozygous individuals. This biological variability may partly explain the differential clinical response to lactose restriction observed in the present study. Similar observations regarding heterogeneous clinical expression among genotype groups have been described, although findings remain inconsistent across populations [28,29]. Previous studies evaluating genotype-specific responses to lactose restriction have reported variable associations between LCT polymorphisms and symptom improvement. Some investigations suggested that genetic lactase non-persistence is strongly associated with dietary response, whereas others demonstrated weaker genotype–phenotype correlations and emphasized the role of intestinal microbiota, lactose exposure, and visceral sensitivity in modulating clinical outcomes. The findings of the present study further support the concept that lactose intolerance represents a biologically and clinically heterogeneous condition [1,3,6].

From a clinical perspective, the present findings support the integration of LCT genotyping as a complementary tool in the diagnostic evaluation of adults with suspected lactose intolerance, particularly in patients with persistent or inconclusive symptoms. Genetic testing should not replace clinical assessment but may contribute to a more individualized diagnostic approach when interpreted together with clinical history, symptom evaluation, and dietary response. Such an integrated strategy may help reduce unnecessary long-term dietary restrictions and support more personalized nutritional counseling.

The unexpectedly higher response rate observed in CT carriers should be interpreted with caution. This finding may reflect several factors, including differences in lactose exposure, biological heterogeneity in lactase activity, differences in dietary adherence, gut microbiota composition, or unmeasured confounding variables influencing symptom perception [30,31]. Similar inconsistencies between genotype and clinical phenotype have been previously reported, with studies highlighting the limited predictive value of genetic testing alone for symptom severity in lactose intolerance [19,32,33,34]. In addition, several potential confounding factors were not systematically assessed because of the retrospective design of the study. Conditions such as irritable bowel syndrome, secondary lactose intolerance resulting from underlying gastrointestinal diseases, differences in gut microbiota composition, and other functional or organic gastrointestinal disorders may have contributed to symptom perception and clinical response independently of LCT genotype. Therefore, these factors should be considered when interpreting the present findings and warrant further investigation in prospective studies using standardized clinical assessment protocols.

These results also underscore the complex relationship between genetic lactase non-persistence and clinically expressed lactose intolerance. Not all individuals with lactase non-persistence develop symptoms, and symptom severity may vary substantially depending on dietary exposure, visceral sensitivity, and intestinal microbiome composition. Previous research has similarly demonstrated inconsistent correlations between LCT genotype and gastrointestinal symptoms, supporting the notion that lactose intolerance is a multifactorial condition rather than a purely genetically determined phenotype [2,35,36,37,38].

An important distinction should be made between lactose malabsorption and lactose intolerance. Genetic testing primarily identifies a predisposition to lactase non-persistence and subsequent lactose malabsorption, whereas the clinical manifestations evaluated in the present study reflect lactose intolerance, a symptomatic condition influenced by multiple biological and environmental factors. Consequently, genetic findings should not be interpreted as direct predictors of symptom burden, but rather as one component of a broader clinical assessment.

The presence of a lactase-persistence-associated genotype does not, however, exclude the presence of gastrointestinal symptoms attributed by patients to lactose consumption. In the present cohort, symptoms among CT and TT carriers may reflect the nonspecific nature of gastrointestinal complaints and the possible contribution of functional gastrointestinal disorders, including irritable bowel syndrome, as well as individual differences in symptom perception and dietary factors. Because the retrospective design did not include a blinded lactose challenge or systematic diagnostic assessment for irritable bowel syndrome and other functional gastrointestinal disorders, the direct causal relationship between lactose ingestion and the reported symptoms cannot be established. Accordingly, the observed clinical response should be interpreted as patient-reported improvement following dietary lactose restriction rather than as definitive evidence of lactose-specific symptom causality.

An interesting observation was that a small proportion of TT individuals (17.6%) also reported symptom improvement following lactose restriction despite the absence of a genetic predisposition to primary lactose intolerance. Several explanations may account for this finding. First, gastrointestinal symptoms commonly attributed to lactose intolerance are nonspecific and may overlap with those observed in functional gastrointestinal disorders, particularly irritable bowel syndrome. Second, reducing dairy intake may inadvertently decrease the consumption of other fermentable dietary components that contribute to bloating and abdominal discomfort. Finally, placebo effects, variations in symptom perception, and individual dietary behaviors may also influence the reported clinical outcomes. These observations further support the concept that symptom improvement following dietary restriction cannot be explained solely by LCT genotype and should be interpreted within a broader clinical context.

Recent developments in precision nutrition and nutrigenomics have increasingly highlighted the relevance of genotype-guided dietary interventions in gastrointestinal disorders. Within this framework, lactose intolerance represents a clinically relevant model for exploring the interaction between genetic predisposition and individualized dietary response. The present findings support the concept that genetic variability may contribute to differences in treatment response, although clinical outcomes are likely influenced by multiple additional biological and environmental factors [12,16].

Moreover, growing evidence suggests that gut microbiota composition may modulate lactose metabolism and symptom generation, potentially contributing to variability in lactose tolerance even among individuals with similar genetic backgrounds. This emerging perspective further supports the need for integrated clinical assessment strategies that combine genetic testing with symptom evaluation and dietary response in order to achieve a more personalized approach to patient management [26,39].

The present findings may also have relevant implications for nutritional counseling and long-term dietary management. In clinical practice, many patients initiate restrictive lactose-free diets based primarily on subjective symptom perception, often without objective diagnostic confirmation. While lactose restriction may improve symptoms in selected individuals, unnecessary long-term exclusion of dairy products may contribute to inadequate calcium and vitamin D intake, potentially affecting bone health and overall nutritional status. In this context, integrating genetic information with clinical assessment may support more individualized dietary recommendations and help avoid excessive dietary restrictions in patients without clinically relevant lactose intolerance [1,3,40].

An additional aspect worth considering is the potential psychosocial impact associated with chronic gastrointestinal symptoms attributed to lactose intolerance. Recurrent bloating, abdominal discomfort, and unpredictable digestive symptoms may negatively influence eating behavior, social interactions, and overall quality of life. Some patients may progressively develop restrictive dietary habits or food-related anxiety, particularly in the absence of a clear diagnosis or appropriate nutritional counseling. Consequently, a more comprehensive diagnostic approach integrating clinical evaluation and genetic testing may contribute not only to symptom management, but also to improved patient reassurance and long-term dietary adherence [1,3].

The observed heterogeneity in clinical response among genotype groups also highlights the importance of considering lactose intolerance within a broader biopsychosocial and nutritional framework. Gastrointestinal symptom perception may be influenced not only by lactase activity, but also by dietary habits, intestinal microbiota composition, psychological factors, visceral hypersensitivity, and coexisting functional gastrointestinal disorders. This complex interaction between genetic and non-genetic factors may partly explain why genotype alone cannot fully predict symptom severity or response to dietary intervention. Therefore, a multidimensional clinical approach remains essential in the evaluation and management of patients with suspected lactose intolerance [1,6,41].

Although gastrointestinal symptoms are frequently attributed to lactose intolerance, alternative gastrointestinal disorders may produce similar clinical manifestations. In the present cohort, positive fecal Helicobacter pylori antigen tests and positive celiac disease screening results were identified in only a small proportion of patients. While these conditions were unlikely to account for the majority of symptoms observed, their presence underscores the importance of differential diagnosis in patients presenting with bloating, abdominal pain, and bowel habit disturbances. These findings further support the concept that gastrointestinal symptom generation is multifactorial and cannot always be attributed exclusively to lactose intolerance.

From a broader medical assessment standpoint, integrating genetic data with observed dietary response may contribute to a more structured and objective evaluation of patients. Gastrointestinal symptoms are inherently subjective and may be influenced by reporting variability, psychological factors, and coexisting functional gastrointestinal disorders [42,43,44]. In this context, combining genotype information with real-world clinical response to lactose restriction may enhance diagnostic precision, particularly in cases with nonspecific or overlapping symptomatology. These findings align with the growing interest in personalized nutrition approaches and the integration of genetic markers into dietary management strategies [45,46].

In addition, the increasing clinical interest in food intolerance-related disorders highlights the importance of developing standardized and evidence-based diagnostic strategies. Combining clinical evaluation, dietary assessment, and genetic testing may contribute to more accurate patient stratification and improve the overall management of individuals presenting with lactose-related gastrointestinal symptoms [1,20].

Future studies should further investigate the mechanisms underlying genotype-specific variability in clinical response to lactose restriction. Prospective longitudinal studies incorporating standardized symptom scoring systems, objective dietary adherence assessment, and gut microbiome analysis may provide additional insight into the complex relationship between genetic lactase persistence, intestinal adaptation, and symptom generation. Moreover, the integration of nutrigenomic and microbiome-related biomarkers may contribute to the development of more personalized and evidence-based dietary management strategies in patients with lactose intolerance [12,16,39,47].

From a public health perspective, the increasing availability of direct-to-consumer genetic testing and growing public interest in food intolerance raise additional challenges regarding interpretation and clinical applicability. Genetic predisposition alone does not necessarily equate to clinically significant lactose intolerance, and inappropriate interpretation of genetic results may lead to unnecessary dietary restrictions. Therefore, healthcare professionals should interpret LCT genotypes within the broader clinical and nutritional context, emphasizing individualized assessment and evidence-based dietary recommendations. These considerations further support the need for balanced integration of genetic testing into routine clinical practice [12,16].

Another clinically relevant consideration is the potential role of patient education in the management of lactose intolerance. Adequate counseling regarding lactose content in foods, individual tolerance thresholds, and balanced dietary alternatives may significantly improve long-term adherence and symptom control. Moreover, patients frequently associate a broad range of nonspecific gastrointestinal symptoms with lactose ingestion, which may contribute to unnecessary dietary restrictions and reduced dietary diversity. Therefore, improving patient awareness and promoting evidence-based nutritional guidance remain important components of individualized clinical management strategies in lactose intolerance [1,3].

Several limitations should be acknowledged. First, the retrospective observational design may have introduced selection and information bias, as only patients with complete clinical and genetic records were included in the analysis. Second, symptom improvement was assessed based on patient self-report during routine clinical follow-up rather than through validated symptom severity questionnaires or standardized lactose challenge testing. Consequently, the evaluation of clinical response may have been influenced by subjective symptom perception and reporting variability. Third, adherence to the lactose-free diet was assessed during routine clinical consultations and was not objectively quantified using structured dietary assessment tools. Consequently, variations in dietary compliance may have influenced the observed clinical response. Furthermore, although alternative causes of gastrointestinal symptoms were investigated in some patients, potential confounding factors such as functional gastrointestinal disorders, gut microbiota composition, and other dietary influences could not be systematically evaluated. In addition, although fecal *Helicobacter pylori* antigen testing and Celikey^®^ screening were performed in selected patients according to clinical judgment, the small number of positive cases makes it unlikely that these conditions substantially influenced the overall results. Although multivariate and stratified analyses were performed, residual confounding cannot be excluded. In addition, the relatively small TT subgroup resulted in wider confidence intervals in the logistic regression analysis, which may have reduced the precision of the estimated odds ratios despite the consistency observed across different statistical approaches.

Furthermore, placebo effects, spontaneous symptom variability, and concurrent dietary or lifestyle modifications cannot be excluded as contributors to the observed clinical improvement.

Despite these limitations, the relatively large sample size, the availability of both genetic and clinical follow-up data, and the consistency of findings across multiple analytical approaches strengthen the validity of the results. The observed association between LCT genotype and clinical response to lactose restriction provides additional evidence supporting a role for genetic factors in modulating symptom outcomes while also highlighting the complex relationship between genetic predisposition, lactose malabsorption, and clinically expressed lactose intolerance in real-world settings. Future prospective studies incorporating validated symptom assessment tools, objective dietary adherence measures, and a comprehensive evaluation of potential confounding factors are warranted.

## 5. Conclusions

LCT genotype was significantly associated with patient-reported clinical response to a lactose-free diet in adults evaluated for suspected lactose intolerance. Heterozygous CT individuals demonstrated the highest rate of symptom improvement, followed by CC and TT genotypes.

These findings suggest that genetic variation may contribute to differences in symptom response to dietary lactose restriction and support the use of genotypic information as a complementary component of clinical assessment. However, the observed variability in clinical outcomes across genotype groups highlights the multifactorial nature of lactose intolerance and indicates that genetic predisposition alone does not fully explain symptom expression or dietary response. Therefore, genetic testing should be interpreted as a complementary component of the diagnostic evaluation rather than a standalone method for diagnosing lactose intolerance and should always be considered together with clinical history, symptom assessment, and dietary evaluation.

Further prospective studies incorporating validated symptom assessment tools and objective measures of dietary adherence are warranted to better define the clinical utility of genotype-guided management strategies in patients with suspected lactose intolerance.

## Figures and Tables

**Table 1:** Baseline characteristics of the study population (*n* = 538).

Variable	Value
Age (years), mean ± SD	39.8 ± 11.0
Sex, *n* (%)	
Male	175 (32.5)
Female	363 (67.5)
Residence, *n* (%)	
Urban	456 (84.8)
Rural	82 (15.2)
Genotype distribution, *n* (%)	
CC	381 (70.8)
CT	123 (22.9)
TT	34 (6.3)

**Table 2:** Baseline gastrointestinal symptoms according to LCT genotype.

Symptom	Total *n* (%)	CC *n*	CT *n*	TT *n*
Abdominal bloating	538 (100.0)	381	123	34
Abdominal pain	252 (46.8)	185	51	16
Bowel habit disturbances (diarrhea and/or constipation)	124 (23.0)	83	32	9

**Table 3:** Additional gastrointestinal investigations.

Test	Total Positive	CC	CT	TT
Fecal *Helicobacter pylori* antigen	19	14	5	0
Celikey screening test	5	4	0	1

**Table 4:** Clinical response to lactose-free diet according to LCT genotype.

Genotype	Improved	Partial/No Improvement
CC	167 (43.8%)	214 (56.2%)
CT	87 (70.7%)	36 (29.3%)
TT	6 (17.6%)	28 (82.4%)

χ^2^ test, *p* < 0.001.

**Table 5:** Stratified analysis of the association between LCT genotype and clinical response using the Mantel–Haenszel method.

Stratum	a	b	c	d
Male < 38	16	39	0	2
Male 38–52	17	48	1	2
Male > 52	17	29	0	5
Female < 38	38	77	1	8
Female 38–52	39	66	0	9
Female > 52	43	75	2	5

Legend: a = genotype-positive with symptom improvement; b = genotype-positive without symptom improvement; c = genotype-negative with symptom improvement; d = genotype-negative without symptom improvement. Mantel–Haenszel pooled odds ratio (OR_mh_) = 3.95. χ^2^MH = 6.70; *p* = 0.0097.

## Data Availability

The data presented in this study are available on request from the corresponding author due to privacy restrictions concerning individual patient data.
